# Spectral Content Effects Study in Non-Contact Resonance Ultrasound Spectroscopy

**DOI:** 10.3390/s25010265

**Published:** 2025-01-05

**Authors:** Muhammad Tayyib, Linas Svilainis

**Affiliations:** Department of Electronics Engineering, Kaunas University of Technology, 51368 Kaunas, Lithuania; linas.svilainis@ktu.lt

**Keywords:** air-coupled ultrasound, spread-spectrum signals, spectral losses compensation, resonance ultrasound spectroscopy

## Abstract

The application of spread-spectrum signals (arbitrary pulse width and position (APWP) sequences) in air-coupled resonant ultrasound spectroscopy is studied. It was hypothesized that spread-spectrum signal optimization should be based on te signal to noise ratio (SNR). Six APWP signal optimization criteria were proposed for this purpose. Experimental measurements were conducted using a thin polycarbonate sample using two standard spread-spectrum signals, linear and nonlinear frequency modulation, together with six optimized APWP signals. It was found that the performance of APWP signals derived from linear frequency modulation was better. The two best performing optimization criteria are SNR improvement on a linear scale with the SNR as an additional weight and energy improvement on a dB scale. The influence of spectral coverage on measurement errors was evaluated. It was found that it is sufficient to cover the sample resonance peak and the valley. The lowest error rates for density, 3%, and for thickness, 3.5%, were achieved when the upper valley was covered. For velocity, the best result, 5%, was achieved when the lower valley was covered. The lowest error rate for attenuation, 3.8%, was achieved in the case when both valleys were covered. Yet no significant performance degradation was noted when a whole −30 dB passband was covered.

## 1. Introduction

The use of ultrasonics is encouraged due to low-cost equipment, non-ionizing radiation and small size. One example could be resonant ultrasound spectroscopy (RUS) which uses the eigenfrequencies of a sample to nondestructively characterize material properties [1,2,3]. It was applied for the characterization of thermally conditioned highly explosive materials [4], allowing one to investigate the thermal characteristics of aging-related effects and binderized plastic bonds between the explosive materials. RUS has proven useful in the study [5] of micro-electromechanical systems (MEMSs) for characterizing silicon structures. Conventional RUS data are collected in contact with a sample. However, non-contact measurement is a desired mode. Publication [6] proposed a non-contact, nonlinear RUS version of the method of evaluation of additively manufactured stainless steel parts, allowing one to evaluate the samples’ nonlinear response. Publication [7] proposed a method for simultaneously estimating sample thickness and velocity by using the delay in the pulse after sample insertion and the distance between multiple reflections in sample thickness. Laser excitation was proposed in [8] and spectroscopy was carried out on the received signal, but the signal was received by a contact transducer.

Another option is to use the thickness resonance of the sample in air [9,10,11,12,13,14,15,16,17,18,19,20,21]. This option usually uses non-contact resonant ultrasound spectroscopy (NC-RUS). The main advantage is that measurement is carried out without physical contact with the sample; in cases of completely overlapping internal reflections, both the thickness and velocity of the sample are estimated simultaneously, together with ultrasound attenuation and density.

The advantages for plant leaves and samples where wetting may alter the physical properties have been demonstrated in [10,11,19]. Wideband air-coupled ultrasound has been employed to measure the transmission coefficient of open-cell reticulated solid foam using spatial normalization techniques [14], and the complex structure of solid foams was studied using phase spectroscopy. The use of air-coupled ultrasound through transmission ultrasonic spectroscopy was also applied for the characterization of mineral paper [15], where the spectral response of the thickness and the plate resonance were used to determine the attenuation and velocity coefficients for shear and longitudinal wave velocities within the paper sheet. The application of NC-RUS for the investigation of the textual properties of pork burger patties was also achieved [16]; burger thickness was accurately estimated by measuring the lean fraction’s texture and their combination was measured based on the structure and composition of the material using airborne ultrasonics. NC-RUS has also been utilized for the evaluation of ultrasound velocity and thickness in porous membranes, vegetable samples and composite plates [17]. Another application of NC-RUS, demonstrated in [18], is in the study of ferroelectret sample properties, such as film thickness, velocity, density and attenuation.

NC-RUS faces several challenges, such as a limited bandwidth, high attenuation in air, acoustic impedance mismatch, low transmission efficiency and the dispersion of beams; thus, it is more suitable for thin-sample measurements. For instance, air-coupled transducers have been used for leaf measurements [10,20], but limitations persist due to the restricted bandwidth, a poor SNR and the fact that the resonance frequency might fall outside the passband region. Usually, the solution is to use several pairs of transducers [11,18,19,20], nonlinear ultrasound [21] or laser excitation and reception [8] to cover a sufficient bandwidth. Ultrasound in such applications is produced using a single pulse for excitation [11], which is inefficient because a wider bandwidth requires shorter pulse durations, leading to reduced energy and a lower SNR; most of the energy is concentrated at lower frequencies, causing a lower SNR at higher frequencies. Furthermore, the limited bandwidth of ultrasonic transducers results in a low SNR at the edges of the passband.

The spectral losses can be compensated by using amplitude-modulated (AM) signals [22], yet these require complex electronics (DAC and analog power amplifier) which are inefficient power-wise. Spread-spectrum (SS) [23] signals offer an improved SNR and bandwidth and are efficient energy-wise. Nonlinear frequency modulation (NLFM) SS signals offer the advantage of having a programable spectral shape [24,25]. The rectangular version (unipolar or bipolar) of such signals does not require analog electronics, so more compact and energy-efficient electronics can be used. However, rectangular envelopes introduce significant spectral ripples. Envelope apodization can solve the problem but this again requires complex electronics. Better results can be achieved using arbitrary pulse width and position (APWP) sequences [26]. It has already been demonstrated that spectral losses can be compensated by optimizing the APWP signal for spectral flatness, which is achieved by pushing the energy from the passband into transition bands to increase the bandwidth [27,28]. Yet it remains unclear as to what are the optimization requirements in the case of NC-RUS.

The present work aims to derive and compare several spectral shape optimization criteria and analyze what the spectral coverage should be relative to the transducer passband and sample resonances in order to ensure the lowest measurement error. The analysis is based on the bias errors of four inverse solution parameters: sample thickness, density and ultrasound velocity and attenuation.

## 2. Air-Coupled Resonance Ultrasound Spectroscopy

**Figure 1:**
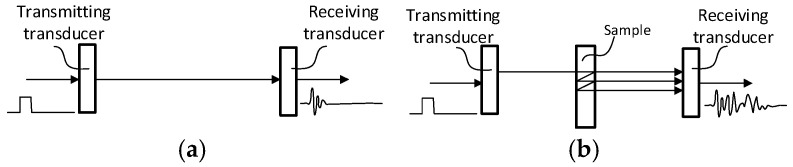
NC-RUS measurement setup: (**a**) calibration and (**b**) sample.

The air-coupled NC-RUS approach is described in [12,13,29]. Two transducers mounted at a known distance D are configured with one as the transmitter and the other as the receiver. The transmission response requires two measurements: (i) a calibration measurement, when the path between the transducers is free from obstacles, and (ii) a sample measurement, with the sample inserted between the transducers (Figure 1).

The difference between sample and calibration signals is the propagation within the sample effects. Then, sample parameters can be extracted from the transmission response which is obtained by calculating the ratio between the sample and calibration spectra.

A slightly different approach, adopted from [12], is used in the present study. The difference in approach here is that fitting is carried out in the time domain. Fitting in the time domain requires gating of the received signal. Convolution with the *sinc* function causes ringing in the transmission spectra if the gate cuts out a portion of the received signal. Then, fitting quality is significantly reduced. This is the reason why a single pulse is used in NC-RUS. Fitting in the time domain allows us to gate just the portion of the signal without adverse effects. Long APWP excitation signals are incorporated in the present case, so such an advantage is important here.

The sample signal *S_mod_* is derived from the measured calibration signal *R* using the transmission model *T*:

(1) $$S_{mod}\left(\omega ,y,x\right)=T(\omega ,y,x)\cdot S_{cal}(\omega )\cdot e^{j\omega \frac{h}{c_{air}}},$$

where *T*(*ω*,**y**,**x**) is a transfer function which depends on the air parameter (ultrasound velocity and density), i.e., vector **x**, and sample parameters (thickness, density, ultrasound velocity and attenuation), i.e., vector **y**; *S_cal_*(*ω*) is the calibration signal, *ω* = 2*πf* is the angular frequency, *h* is the thickness of the sample and *c_air_* is the velocity of ultrasound in air.

The transmission model, described in Berkhovskikh’s study [30], is used for the transmission of ultrasound through a sample with acoustic impedance *Z_s_* and air impedance *Z_air_* in the frequency domain:

(2) $$T\left(\omega \right)=\frac{-Z_{air}Z_{s}}{{-2Z}_{air}Z_{s}\cos\left(k^{\prime }h\right)+j(Z_{air}^{2}+Z_{s}^{2})sin(k^{\prime }h)},$$

The complex wave number *k*′ is defined as follows:

(3) $$k^{\prime }=\frac{\omega }{c_{s}}-j\alpha ,\;\alpha =\alpha_{0}\cdot {\left(f/f_{0}\right)}^{n_{a}},$$

where *c_s_* is the ultrasound velocity, α is the attenuation of the sample [29], *f*_0_ is the center frequency of the transducer (normalization frequency) and *n_a_* is the power law for attenuation which is frequency-dependent. The acoustic impedance is calculated as follows:

(4) $$Z_{air}=c_{air}\cdot \rho_{air},\;Z_{s}=c_{s}\cdot \rho_{s},$$

where *c_s_* is the ultrasound velocity in the sample, and *ρ_air_* and *ρ_s_* are the density of air and the sample, respectively.

The sample parameter vector, **y** = (α_0_, *c_s_*, *ρ_s_*,*h*, *n_a_*), is obtained via the inverse solution of (1), which minimizes the difference between the measured *s_meas_* and the modeled *s_mod_* signals in the time domain:

(5) $$y=argmin\sum_{i=1}^{N}{\left(\frac{s_{mod}{\left(x,y\right)}_{i}-s_{imeas}}{s_{iRS}}\right)}^{2}.$$

**Figure 2:**
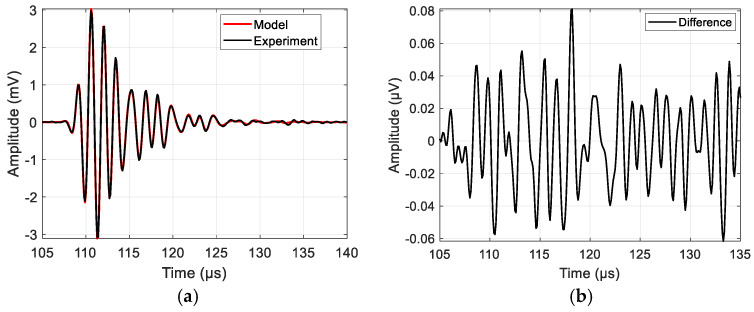
NC-RUS fitting result for sample signal when pulse excitation is used: (**a**) model and measurement signal comparison; (**b**) difference between signals obtained via subtraction of model signal from experimental one.

An example of the resulting model signal compared to the measured one is presented in Figure 2.

A fairly good match is obtained. The remainder of the signal after subtraction is plotted to the right in order to demonstrate the goodness of fit. The reason for such results is a high SNR (good sample resonance peak match to transducer passband, low sample attenuation and sample impedance close to that of air). A higher SNR and a broader bandwidth are required to make such a measurement system fit for a broad range of samples. Therefore, SS signals are used in further investigations. The aim is to derive optimal APWP optimization criteria and bandwidth coverage relative to the available −30 dB passband.

## 3. APWP Signal Derivation

**Figure 3:**
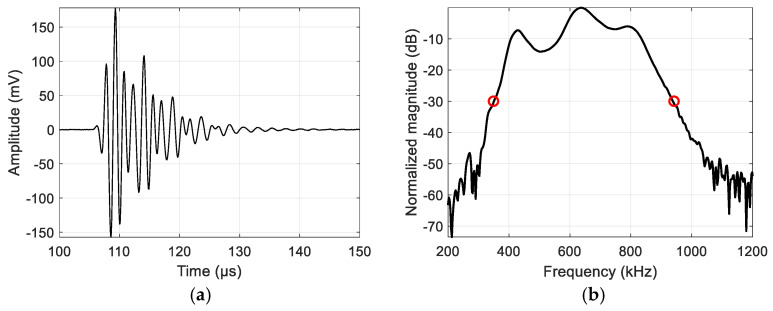
Received signal after pulse excitation: (**a**) time domain; (**b**) normalized in spectral domain (circles: −30 dB bandwidth).

The calibration signal spectrum in the case of single-pulse excitation is presented in Figure 3 to demonstrate the transducer bandwidth. Two air-coupled 650 kHz ultrasonic transducers produced by CSIC, Spain, were used.

It can be seen that the bandwidth is already broad: a −30 dB bandwidth covers a 340–940 kHz range (98% of center frequency). The bandwidth was measured at −30 dB because this is the level where the SNR is still sufficient for reliable processing. The above-mentioned frequency range was used in further investigations, assuming that a good SNR cannot be achieved beyond this range even with properly derived spectral compensation.

### 3.1. Noise

It is important to note that it is the SNR, not just signal energy, that determines the estimation accuracy. Consequently, signal optimization was based on the SNR, an improvement over previous works [27,28] where just signal energy was considered.

The SNR frequency domain response was obtained as the ratio of signal *S_cal_* to noise *e_n_* voltage spectral densities:

(6) $$SNR=\frac{S_{cal}}{e_{n}}.$$

The noise voltage spectral density (NVSD) AC response was obtained by measuring 10,000 waveforms of the receiving amplifier output with no excitation signal. The resulting waveforms were transformed into the frequency domain, averaged power-wise and normalized to derive the NVSD, *e_n_*:

(7) $$e_{n}(f)=\sqrt{\frac{2\times \sum_{1}^{N}{\left|DFT(s_{RS})\right|}^{2}}{N\cdot fs\cdot M}},$$

where *f_s_* is the sampling frequency, *M* is the number of measured noise waveforms and *N* is the number of samples.

The signal *S_cal_* was obtained as the calibration signal (no sample in between transducers) when the transducer was excited using conventional linear frequency modulation (LFM) with 340 kHz to 940 kHz bandwidth coverage.

With the SNR available, APWP signal derivation was performed.

### 3.2. SNR Equalization

The compensation function ξ was derived from the SNR by using an additional window *W*, which aims to suppress the noise at the edges of the transducer passband (for *f*_0_ = 650 kHz, an attainable bandwidth is between 340 kHz and 940 kHz).

(8) $$\xi =\frac{\left|W(f)\right|}{\left|SNR(f)\right|}.$$

**Figure 4:**
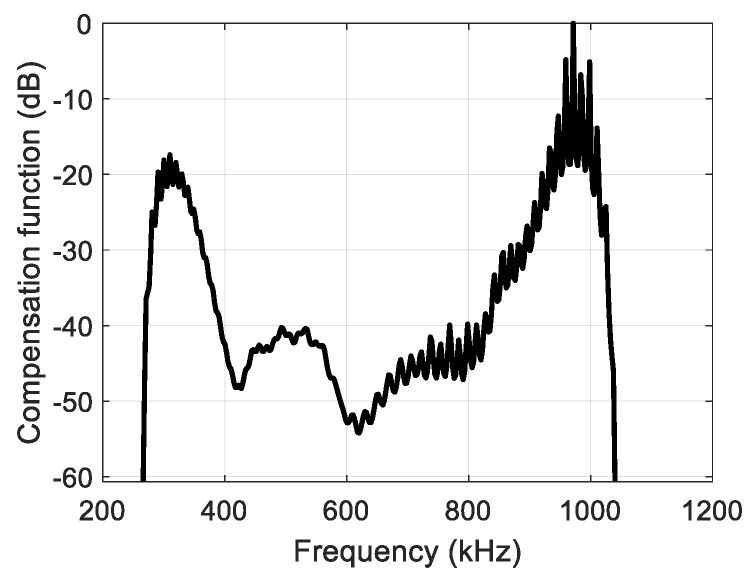
SNR-based compensation function.

The compensation function is shown in Figure 4.

The NLFM signal can be derived from the compensation function. As energy for each spectral component is dependent on the rate of change in the instantaneous frequency, time rate versus frequency is calculated as follows:

(9) $$\tau \left(f^{\prime }\right)=\int_{0}^{f^{\prime }}{\left|\xi (f)\right|}^{2}\cdot df.$$

Inversion of (9) is obtained by interpolation:

(10) $$\tau \left(f^{’}\right)\overset{Interp}{\to }f^{’}\left(\tau \right).$$

Phase *θ* is obtained via the integration of instantaneous frequencies:

(11) $$\theta \left(t^{\prime }\right)=2\pi \int_{0}^{t^{\prime }}f^{\prime }\left(t\right)\cdot dt.$$

Then, the unipolar NLFM excitation signal *s*_TXN_ is calculated as follows:

(12) $$s_{TXN}\left(t\right)=1+sign\left(\sin\theta \left(t\right)\right)/2.$$

But both LFM and NLFM signals have a rectangular envelope which produces large spectral ripples and is not optimal for providing excitation. Furter optimization is carried out using an APWP derivation algorithm.

### 3.3. APWP Derivation for Optimal Spectrum Shaping

APWP signals require optimization [26], which is carried out by adjusting the width and position of the pulses in time. Unipolar signals were used in this investigation to further simplify the requirements for excitation electronics. Every candidate APWP sequence has to be applied in transmission in order to account for the transducers’ and electronics’ AC response and noise. In order to speed up the optimization process, the actual measurement was replaced by passing the candidate through a system mathematical model. The transmission model was derived from the response in Figure 3 and fitted into an 8-th order infinite impulse response (IIR) filter. Noise, colored by using (7), was added after passing the candidate sequence through the system model.

The optimization process of APWP signal includes the adjustment of the width and position of the pulses in time based on selected convergence criteria. Six criteria were proposed: (i) aimed at SNR flatness, (ii) aimed at LFM SNR improvement on a linear scale, (iii) aimed at LFM SNR improvement on a linear scale with the SNR as an additional weight, (iv) aimed at LFM energy improvement on a dB scale, (v) aimed at NLFM SNR improvement on a linear scale and (vi) aimed at NLFM 1/SNR improvement on a linear scale. Each criterion was calculated within the desired bandwidth *f_min_*-*f_max_*.

The compensated SNR_c_ is obtained from the excitation signal *s*_TXN_ generated in the ith iteration. SNR spectral flatness is evaluated within the desired passband *f_min_*-*f_max_:*

(13) $$\varsigma_{APWP}=\sqrt{\frac{\int_{f_{min}}^{f_{max}}{\left({|SNR}_{Ci}\left(f\right)\left|-\right|{\bar{SNR}}_{Ci}\left(f\right)|\right)}^{2}df}{\left|{\bar{SNR}}_{Ci}\left(f\right)\right|}}.$$

The APWP signal obtained from Expression (13) (where *ς* represents the optimized SNR) tends to distribute energy toward the edges of the passband; however, linear frequency modulation (LFM) of the signal instead of NLFM, which tends to have higher energy, could also be explored as it compensates for the higher energy toward the edges, which might provide a better optimized signal.

(14) $$\varsigma_{LFM}=\sum_{f_{min}}^{f_{max}}\left({|SNR}_{LFMi}\left(f\right)|-{|SNR}_{Ci}\left(f\right)|\right).$$

The new optimization criteria were developed using the same settings but changing Expression (13) for (14), which is basically the energy of the signal. There are a few other ideas that could also provide better results; thus, another approach was developed where the compensated SNR was weighted to give more focus on the energy of LFM:

(15) $$\varsigma_{LFMW}=\sum_{f_{min}}^{f_{max}}\frac{\left({|SNR}_{LFMi}\left(f\right)|-{|SNR}_{Ci}\left(f\right)|\right)}{{|SNR}_{Ci}\left(f\right)|}.$$

There was a difference between the linear and dB scale, so by compensating on the dB scale, different results were obtained:

(16) $$\varsigma_{LFMdB}=\sum_{f_{min}}^{f_{max}}\left(20{log}_{10}{|SNR}_{LFMi}\left(f\right)|-{20{log}_{10}|SNR}_{Ci}\left(f\right)|\right).$$

The SNR obtained from the NLFM signals was subtracted from the APWP signal:

(17) $$\varsigma_{NLFM}=\sum_{f_{min}}^{f_{max}}\left(20\log_{10}|SNR_{NLFMi}\left(f\right)\left|-20\log_{10}|SNR_{Ci}\left(f\right)\right|\right).$$

Lastly, instead of using the SNR for NLFM optimization, the noise-to-signal ratio was used, which is basically 1/SNR.

(18) $$\varsigma_{1/NLFM}=\sum_{f_{min}}^{f_{max}}\left(20\log_{10}|1/SNR_{NLFMi}\left(f\right)\left|-20\log_{10}|1/SNR_{Ci}\left(f\right)\right|\right).$$

**Figure 5:**
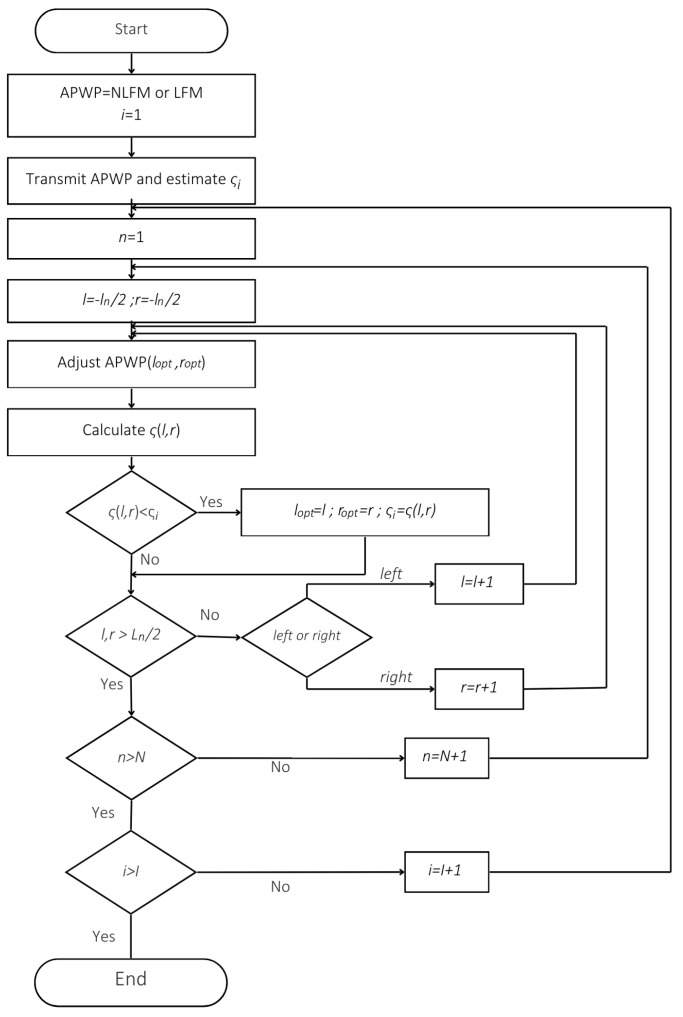
Optimization algorithm for spectral compensation.

The aforementioned criteria consider different aspects of spectra. While (13) aims to achieve spectral flatness, (14), (16) and (17) allow for any spectral shape change that improves the average SNR, (15) tries to account for the spectral shape and (18) is the inverse of the rest, used just to prove that such an approach does not improve the results. The idea that all criteria are used to redistribute the energy suggests that this will improve the measurement results. The optimization algorithm is shown in Figure 5, where for each optimization criterion, *ς* is substituted by (14)–(18).

The algorithm begins with initializing the APWP signal using either NLFM or LFM, with the iteration index set to 1. The process involves transmitting the APWP signal and estimating a performance metric, *ς_i_*. Variables *n*, *l* and *r* are initialized, and adjustments are made to evaluate the function *ς*(*l*,*r*). If the function shows improvement over *ς_i_*, the optimal parameters *l_opt_* and *r_opt_* are updated. Iterative loops ensure that the algorithm explores all possible configurations within predefined limits. As soon as the maximum number of iterations is reached, the process terminates. Usually, 3–5 iterations are sufficient.

**Figure 6:**
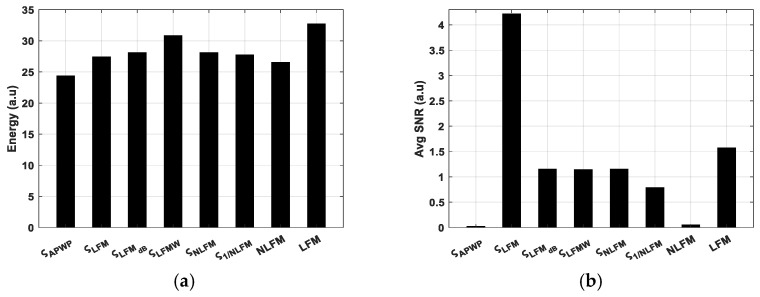
Energy of excitation signal (**a**) compared to average SNR of received sample signal (**b**) for broadest bandwidth coverage, 650 kHz (100% of center frequency).

**Figure 7:**
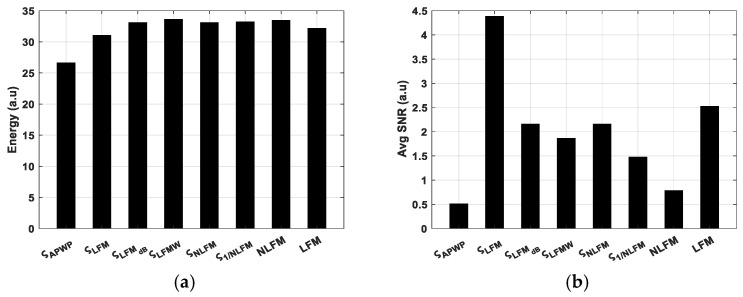
Energy of excitation signal (**a**) compared to average SNR of received sample signal (**b**) for broadest bandwidth coverage, 400 kHz (60% of center frequency).

**Figure 8:**
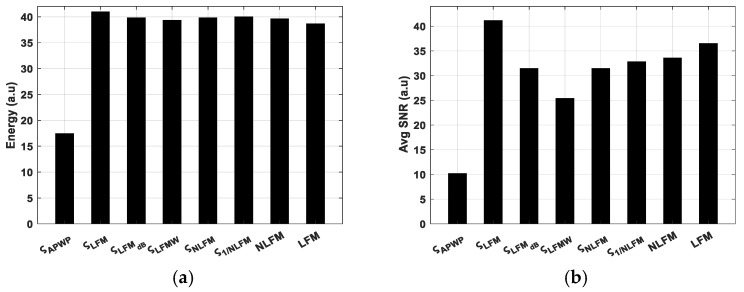
Energy of excitation signal (**a**) compared to average SNR of received sample signal (**b**) for broadest bandwidth coverage, 100 kHz (15% of center frequency).

APWP optimization just slightly modifies the excitation signal energy (refer to Figure 6a,b, as the variation is just ±1 dB for 100% bandwidth coverage. Meanwhile, the average SNR differences are more pronounced (refer to Figure 6b, Figure 7b and Figure 8b).

Nevertheless, as will be seen from the next sections, there is little correlation with the measurement errors.

## 4. Experimental Setup

**Figure 9:**
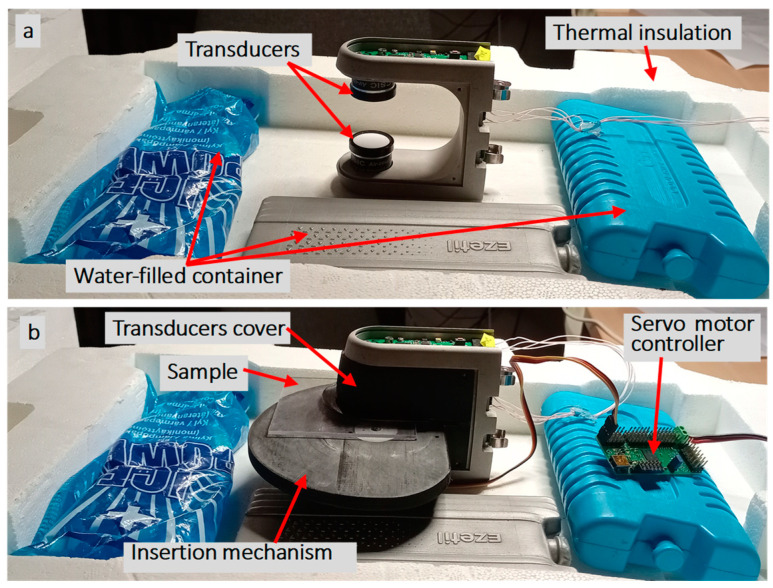
Experimental measurement setup: (**a**) equipment view with insertion mechanism removed; (**b**) insertion mechanism view.

A thin polycarbonate plate with 2.05 mm thickness was used as the sample for the evaluation of the proposed approach. Two air-coupled 650 kHz ultrasonic transducers produced by CISC, Spain, were optimally placed at a known distance of 32 mm from each other (Figure 9). An acquisition system produced by Kaunas University of Technology was used. The electronics included a programmable voltage excitation pulser, capable of APWP pulse train generation, and a programmable gain reception amplifier: 8 dB for calibration measurements and 50 dB for sample measurements. Excitation used 35 V unipolar excitation sequences that were 100 μs long. The ultrasonic system was controlled remotely using Bluetooth communication. All of the other processing steps, inverse solution, optimization and evaluation, were carried out using MATLAB^®^.

The aim of this study was to estimate the sample parameters using NC-RUS. The actual parameters of interest were the attenuation (α), thickness (*h*), ultrasound velocity (*c*) and density (*ρ*) of the sample. Atmospheric pressure, temperature and humidity significantly influence the ultrasound velocity in air [12]. The sample was placed in a thermally insulated chamber with water-filled containers (Figure 9) to reduce the temperature variation.

The setup was left open in laboratory conditions for 24 h so the temperature of the containers could settle. The environmental influence on the air parameter compensation technique, described in [12], was used to improve the measurement accuracy. A servo motor was used to automatically insert and remove samples for calibration and measurement, which were carried out in turns 100 times.

The procedure was carried out the same way for various spectral coverage combinations. Three sets of experiments were designed: (i) concentrated around the resonance peak 620 kHz of the sample; (ii) expanding from the resonance peak toward higher frequencies; (iii) expanding from the resonance peak toward lower frequencies. The bandwidth was increased in 25 kHz steps until the stopband was reached (also corresponded to resonance valleys). Each spectral combination contained 100 calibration–sample pairs which were used in NC-RUS for sample parameter estimation.

## 5. Results

**Figure 10:**
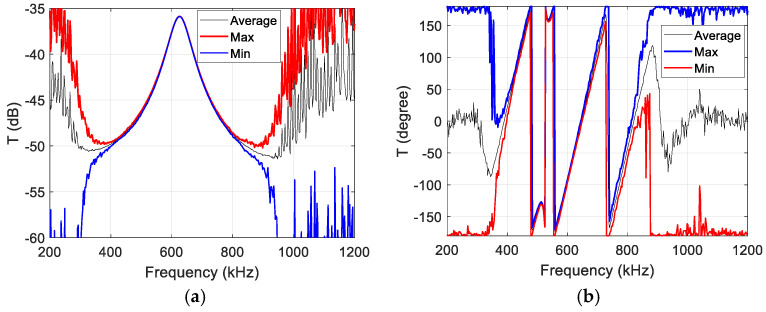
Transmission response of polycarbonate sample: (**a**) magnitude; (**b**) phase.

An example of the resulting transmission response of the polycarbonate sample is presented in Figure 10. The mean, minimum and maximum values are shown to give an impression of where the main variation, caused by noise, occurs.

A total of eight signals were used—LFM, NLFM and six APWP signals—optimized according to (13)–(18). Each signal combination produced 100 results for each step of bandwidth variation; three bandwidth placements were evaluated. The results were processed to obtain the mean and then compared against caliper measurements to obtain the bias error.

### Spectrum Location and Bandwidth Influence

**Figure 11:**
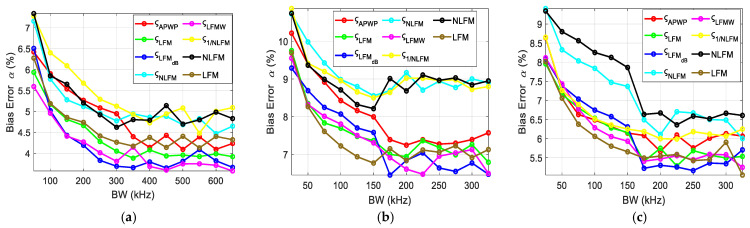
Attenuation bias errors for the polycarbonate sample when the excitation coverage is (**a**) concentrated around the resonance peak, (**b**) expanding from the resonance peak toward higher frequencies and (**c**) expanding from the resonance peak toward lower frequencies.

**Figure 12:**
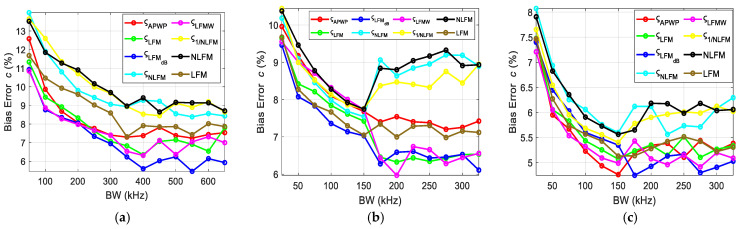
Ultrasound velocity bias errors for the polycarbonate sample when the excitation coverage is (**a**) concentrated around the resonance peak, (**b**) expanding from the resonance peak toward higher frequencies and (**c**) expanding from the resonance peak toward lower frequencies.

**Figure 13:**
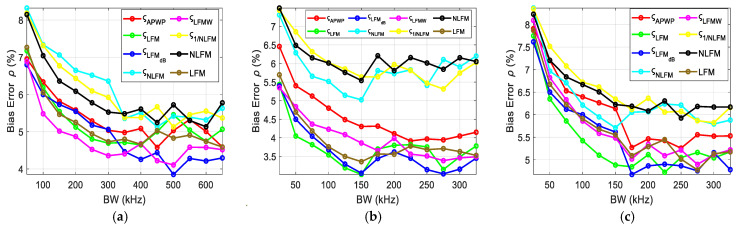
Density bias errors for the polycarbonate sample when the excitation coverage is (**a**) concentrated around the resonance peak, (**b**) expanding from the resonance peak toward higher frequencies and (**c**) expanding from the resonance peak toward lower frequencies.

**Figure 14:**
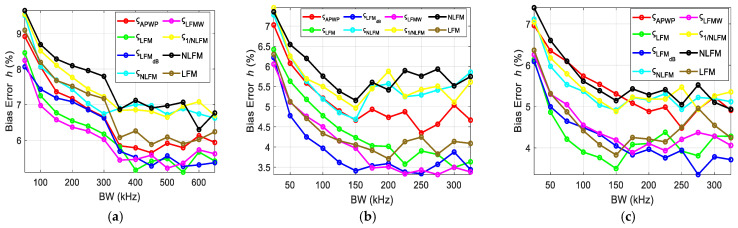
Thickness bias errors for the polycarbonate sample when the excitation coverage is (**a**) concentrated around the resonance peak, (**b**) expanding from the resonance peak toward higher frequencies and (**c**) expanding from the resonance peak toward lower frequencies.

To investigate the influence of the resonance peak and bandwidth of transducer, the scheme proposed was to vary the bandwidth around the resonance peak and observe the relative effect on bias errors. The bias errors for each case were combined and displayed for each parameter, i.e., the ultrasound attenuation *α*_0_ (Figure 11), velocity *c* (Figure 12), density *ρ* (Figure 13) and thickness *h* (Figure 14) of the sample. Bias errors were calculated by taking the average and comparing it to the actual value (*α*_0_ = 23 Np/m, *c* = 2190 m/s, *h* = 2050 µm, *ρ* = 4000 kg/m^3^, [12]) of the polycarbonate sample.

When the bandwidth was narrow, the error rates were high. Once the bandwidth covered the peak and was midway to the valley, the errors became lower, and further bandwidth increases produced less improvement. The best results were obtained for *ς*_LFMdB_, while NLFM performed the worst.

The errors for the ultrasound velocity of the polycarbonate sample are displayed in Figure 12.

The ultrasound velocity bias errors also follow the attenuation trend as these parameters are correlated with each other. The proposed *ς*_LFMdB_-based optimization technique provides the best results among all those compared, while the NLFM signal performs the worst in terms of error performance.

The bias errors for density are displayed in Figure 13.

The bias errors for the thickness of the sample are displayed in Figure 14. The bandwidth is crucial in the NC-RUS investigation as it affects the quality and accuracy of the measurement process. The important thing to understand here is that the bandwidth cannot be selected randomly; moreover, in cases of compensation [27,28], energy from one part of the spectrum is shifted to another part.

The question arises as to which part of the spectrum should the energy be focused on; thus, one assumption could be to focus the energy on the valley as the resonance peak already contains sufficient energy.

The comprehensive bandwidth investigation reveals the importance of both the resonance peak and the valley. From the error estimation (Figure 11, Figure 12, Figure 13 and Figure 14), it is proven that once the resonance peak and halfway to the valley is covered, errors are reduced significantly and stabilize afterwards. Through comparison with other known techniques, i.e., NLFM and LFM, the proposed *ς*_LFMdB_ approach provides significant improvement and error reduction.

## 6. Conclusions

Air-coupled ultrasound resonant spectroscopy demands a wide signal bandwidth and a high SNR, which cannot be attained with pulse excitation. Spread-spectrum signals with a programmable bandwidth allow us to achieve a high SNR and a wide bandwidth simultaneously.

It was demonstrated that APWP signals offer better adherence to spectral requirements. Six APWP signal optimization criteria were proposed. It was found that the performance of APWP signals derived from LFM was better. The two best performing optimization criteria are SNR improvement on a linear scale with the SNR as an additional weight and energy improvement on a dB scale.

The influence of spectral coverage on the measurement errors was evaluated. It was found that it is sufficient to cover the sample resonance peak and the valley. The lowest errors for density, 3%, and for thickness, 3.5%, are achieved when the upper valley is covered. For velocity, the best result, 5%, is achieved when the lower valley is covered. The lowest error for attenuation, 3.8%, is achieved in the case when both valleys are covered. Yet no significant performance degradation is noted if the whole −30 dB passband is covered.

It can be concluded that both the resonance peak and the valley have to be covered by excitation spectra. Once the bandwidth covers the peak and midway to the valley, the errors become low, and further bandwidth increases produce less improvement. It is essential to note that further bandwidth increases are not associated with error increases up to the −30 dB passband (these just stabilize). Then, excitation can be optimized using SNR improvement criteria over the −30 dB passband without accounting for the sample response.

This investigation used polycarbonate samples, but the proposed technique can be extended to other materials, such as metals or composites.

## Data Availability

All relevant data are included in the paper.
